# The effect of characteristics of proteins fed during gestation and lactation on development of metabolic syndrome in dams and male offspring of Wistar rats

**DOI:** 10.1002/osp4.95

**Published:** 2017-03-10

**Authors:** A. Jahan‐mihan, C. A. Labyak, A. Y. Arikawa

**Affiliations:** ^1^ Department of Nutrition and Dietetics University of North Florida Jacksonville FL USA

**Keywords:** Amino acid, fetal programming, metabolic syndrome, protein

## Abstract

**Objective:**

The objective of the study is to examine the role of characteristics of proteins in maternal and weaning diets on risk of metabolic syndrome in male offspring.

**Methods:**

Pregnant Wistar rats were allocated to two groups (*n* = 12) and were fed the AIN‐93G diets based on either intact protein‐based diet (IPD) or mixed amino acid‐based diet (AAD) from day 3 of gestation throughout gestation and lactation. Male offspring were weaned to either an IPD or AAD diet for 18 weeks.

**Results:**

In dams, AAD group had lower body weight in post‐partum period and higher pulse rate compared with IPD group. In pups born to AAD dams, birth weight and body weight were significantly lower, and systolic blood pressure and fasting blood glucose were significantly higher compared with those born to IPD dams. Fat/weight ratio, insulin and homeostasis model assessment of insulin resistance were not influenced by either maternal or weaning diet.

**Conclusions:**

These results indicate that the physico‐chemical structure of proteins fed to dams is important in altering risk factors of metabolic syndrome in the offspring, while weaning diets do not seem to play a role. IPD had more favourable effect than AAD. These results may also indicate that dietary recommendations during development must be updated based on physiological properties of dietary proteins that are beyond their nutritional role.

## Introduction

The prevalence of metabolic syndrome (MS) has reached epidemic proportion 1. According to the National Health and Nutrition Examination Survey, the prevalence of MS in 2010 was 34% 2. The increasing prevalence of abdominal obesity, particularly among female adults has been suggested as a major contributing factor to the MS 2. The role of maternal diet during gestation and the early postnatal period on the development of MS in offspring has been previously demonstrated 3, 4, 5. Dietary proteins fed during gestation, lactation or both influenced the phenotype of offspring in humans and animals 6. Both high‐protein and low‐protein maternal diets showed detrimental effects on body weight (BW), blood pressure, and metabolic and food intake regulatory systems in the offspring 7, 8, 9, 10, 11, 12, 13, 14, 15, 16, 17. We have previously reported that pups born to dams fed a soy protein‐based diet had higher BW, systolic blood pressure (SBP) and diastolic blood pressure (DBP) and plasma insulin at week 14 post‐weaning (PW) compared with those born to dams fed a casein‐based diet 18. Food intake was also higher in offspring born to dams fed soy protein diet in PW period 19. These data indicate that even in a nutritionally balanced diet fed during gestation and lactation, the source of protein can influence the risk of MS in the offspring 18, 19, 20.

In spite of considerable evidence indicating the role of maternal dietary proteins in developmental programming, the underlying mechanisms are still elusive. In adults, dietary proteins elicit a wide range of nutritional and biological functions. They are involved in regulation of food intake, glucose and lipid metabolism, blood pressure, bone metabolism and immune function 8. Clearly, these properties of proteins are far beyond their traditional definition as a source of indispensable amino acids and protein synthesis 8.

Proteins in weaning diets may also interact with maternal diet. Bautista *et al*. showed that offspring born to dams fed a low protein diet and weaned to corn and soy protein had lower weight and body fat percentage compared with those born to dams fed a low protein diet and weaned to a casein‐based diet, indicating that the source of protein in the weaning diet altered the outcomes of maternal protein‐restricted diets 21. The source‐dependent effect of proteins on developmental programming supports the potential role of characteristics of individual proteins in their metabolic and physiologic outcomes. Physico‐chemical properties, digestion kinetics, amino acid composition and bioactive peptides (BAPs) encrypted in amino acid sequences of proteins are proposed mechanisms 8. There is also a line of evidence supporting the source‐dependent effect of proteins consumed during pregnancy and lactation on the health of the offspring. Amino acids have their individual effects during development, in addition to their role in protein synthesis. For example, low protein diets with similar protein content but different amounts of methionine (Southampton diet has higher methionine content compared with Hope farm diet) affect programming of blood pressure differently 6. Adding glycine to the Southampton diet, which reduces plasma homocysteine, leads to normalization of blood pressure. This may suggest that the methionine load is a contributor to increased blood pressure in offspring 22. Moreover, taurine supplementation can regulate insulin secretion and promote insulin sensitivity. Taurine can also ameliorate fructose‐induced hyperglycemia, hypertension and hepatic steatosis in pregnant and non‐pregnant rats 23.

Many physiological functions of proteins are attributed to their BAPs 24. For example, BAPs inhibit angiotensin converting enzyme, which lowers blood pressure in experimental animals 25. Casomorphins are one of the major groups of BAPs that are abundant in casein and affect food intake regulation, gastrointestinal (GI) movement and plasma insulin concentrations 26, 27, 28. However, whether BAPs contribute to the effect of maternal proteins on phenotype of offspring is still elusive.

Lastly, amino acid kinetics has also been suggested as a reason for variations in their postprandial metabolic effects. For example, casein is classified as a slow protein leading to delayed amino acid delivery to the gut, because it precipitates under the acidic gastric pH, while whey remains soluble and rapidly passes through the stomach leading to a faster delivery of amino acids to the circulation. Therefore, whey protein ingestion results in larger increases in post‐meal aminoacidemia than casein and leads to a higher protein synthesis, whereas casein has a greater effect on reducing protein breakdown 8.

The primary objective of this study was to examine the hypothesis that characteristics of proteins (intact or amino acid mixture) fed during gestation and lactation play a role on the development of risk factors of MS in the offspring. Additionally, to examine the potential main and interactive effects of maternal and weaning diets, our second objective was to determine the effect of characteristics of proteins of the weaning diet on the outcomes of the maternal diet in the offspring.

## Experimental methods

### Animals and diets

First‐time pregnant Wistar rats were received at day 3 of gestation (Charles River, NC, USA) and housed individually in ventilated plastic cages with bedding at 22 ± 1 °C and 12‐h light–dark cycle (lights off at 0900 to 2100 h). The diets were provided *ad libitum* in glass jars. All rats had free access to water throughout the experiments.

Diets (AIN‐93G) were purchased from Dyets (Dyets Inc. Bethlehem, Pa, USA). The composition (per kilogram diet) of the diets is illustrated in Table 1. The amino acid composition of the intact protein (casein) and the amino acid mix are illustrated in Table 2. The protocol was approved by University of North Florida Institutional Animal Care and Use Committee.

**Table 1:** Composition of intact protein‐based and amino acid‐based AIN‐93G diets

	Intact casein diet	Amino acid diet
Cal	3,597	3,811
	g kg^−1^	
Casein	200	0
Amino acid mix	0	180
Sucrose	100	100
Soybean oil	70	70
*t*‐Butyhydroquinone	0	0.014
Cornstarch	397.5	399.9
Dyetrose	132	145
Cellulose	50	50
Mineral mix	35	35
Sodium bicorbonate	0	7.4
Vitamin mix	10	10
Choline bitartrate	2.5	2.5
l‐Cystine	3	0

**Table 2:** Amino acid composition of intact protein‐based and amino acid‐based AIN‐93G diets

	Intact casein diet	Amino acid diet
	g kg^−1^	
Alanine	4.7	4.5
Arginine	6.5	6.3
Aspartic acid	11.7	11.3
Cystine	3.7†	3.7
Glutamic acid	37.5	36.2
Glycine	3.2	3.1
Histidine	4.7	4.5
Isoleucine	8.6	8.4
Leucine	15.9	15.3
Lysine	13.3	16.1
Methionine	4.7	4.5
Phenylalanine	9.0	8.7
Proline	21.1	20.4
Serine	9.7	9.4
Threonine	6.8	6.6
Tryptophan	2.2	2.1
Tyrosine	9.6	9.2
Valine	10.3	9.9

The amino acid content of the diets is calculated based on purity of the sources (87% and 99% for casein and amino acid mix, respectively).

† The addition of the free amino acids (l‐cystine) to the intact protein‐based diet is included.

### Experimental design

Newly pregnant Wistar rats (*n* = 24) were received at the third day of pregnancy and were allocated to two groups (*n* = 12 per group) and received either an amino acid‐based diet (AAD) or an intact protein‐based diet (IPD) during pregnancy and lactation. BW of the dams was measured weekly. BW of the pups was measured at birth (day 1, after litters were culled to 10 pups per dam) and on days 7, 14 and 21. At weaning (day 21 of age), one male offspring from each dam on each maternal diet group was assigned to either the AAD or IPD (*n* = 12 per group). BW was measured weekly for 18 weeks after weaning. SBP and DBP, pulse rate, fasting blood glucose (FBG) and blood glucose (BG) response to a glucose load were measured at weeks 4, 8, 12 and 16 PW for the offspring and were measured at week 6 PW for the dams. Fat pad mass was measured at killing at week 6 PW for the dams and at week 18 PW for offspring. Plasma glucose and insulin were measured at birth, weaning and at week 18 PW for the offspring and were measured at week 6 PW for the dams.

### Glucose tolerance test

Rats were fasted overnight for 12 h. Blood samples were drawn from the tail vein at fasting and at 15, 30 and 60 min after a glucose administration (0.375 g glucose per mL, 5 g glucose per kg BW) 18.

### Blood pressure

Systolic blood pressure and DBP were measured by a non‐invasive tail‐cuff method (optical plethysmography) using a tail manometer tachometer system (BP‐2000, Visitech system; Apex, NC, USA). Rats were restrained in holders on a constant warm platform (30 °C). They were adapted daily to the procedure for 5 d. On the day of measurement, five mock measurements preceded a series of ten measurements that were used to calculate the average as reported previously 18.

### Blood glucose

Tail vein glucose concentration was assayed using a handheld commercial glucometer (Contour ® Next Blood Glucose Meter, Bayer Healthcare LLC, Mishawaka, IN, USA) using test strips. The accuracy and variance of the glucometer and test strips were examined by applying control solutions (levels 1 and 2) provided by the manufacturer (Bayer, Bayer Healthcare LLC, Mishawaka, IN, USA) 18.

### Blood collection

As previously described 18, trunk blood was collected in chilled vacutainer tubes (BD, Franklin Lakes, NJ, USA) containing EDTA + Trasylol ® (Bayer AG, Leverkusen, Germany) solution (10% blood volume, 5 × 10^8^ IU L). Thereafter, blood samples were centrifuged at 3,000 *g* and 4 °C for 10 min. Plasma was separated and immediately stored at −70 °C.

### Hormone assays

Plasma insulin concentrations were measured using enzyme‐linked immunosorbent assay (catalogue no. 80‐INSRT‐E01, Alpco Diagnostics, Salem, NH, USA) with assay sensitivity of 0.124 ng mL^−1^.

### Body composition

Fat mass and lean mass were measured right after killing at weeks 6 and 18 PW for dams and pups, respectively. Fat mass was measured by dissection of extracted abdominal, epididymal and perirenal fat 18.

### Statistical analyses

The effect of the maternal and weaning diets and their interactions on BW, glucose response, SBP and DBP was analysed by two‐way analysis of variance. When repeated measures were made over time on BW, food intake, SBP, DBP, pulse, BG response and FBG, the PROC MIXED procedure was used with maternal diets, weaning diets and time as main factors. When interactions were statistically significant, a one‐way analysis of variance followed by post hoc Tukey's test was conducted to evaluate treatment effects. The effects of the maternal diets on plasma measures were compared by using a Student's unpaired *t*‐test. BG response was calculated as the total incremental area under the curve (*t*AUC) of the BG concentration over 1 h after receiving glucose administered for the glucose tolerance test. The homeostasis model assessment of insulin resistance index was calculated as fasting glucose multiplied by fasting insulin divided by 22. Data are expressed as means with standard errors. Statistical significance was defined at *P* < 0.05. All analyses were conducted using sas (version 9.4; SAS Institute, Cary, NC, USA).

## Results

### Dams

**Figure 1:**
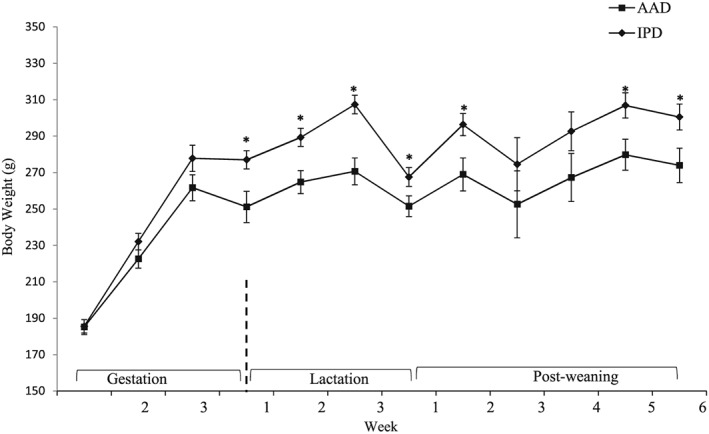
Effect of maternal diet on dams' body weight (*n* = 12 per group). Data are means ± standard error of the mean; body weight was analyzed by one‐way analysis of variance. *P < 0.05; AAD, amino acid‐based diet; IPD, intact protein‐based diet.

Body weight was affected by the diet after parturition, during lactation and at weeks 2, 5 and 6 PW and was higher in dams fed the IPD (*P* < 0.05) (Figure 1). However, no difference in fat/weight percentage at week 6 PW was observed (Table 3). No effect of the diet on SBP and DBP was observed (data not shown), but pulse rate was higher in the AAD group compared with the IPD group (443.64 ± 9.79 vs. 412.99 ± 8.49, respectively) (*P* < 0.005). No effect of the maternal diet on FBG, BG response, homeostasis model assessment of insulin resistance or insulin/glucose ratio was observed (Table 4).

**Table 3:** Effect of maternal diet on body weight, fat and fat% in dams at week 9 post‐partum and offspring at birth, weaning and at week 18 post‐weaning

Maternal diet	IPD	AAD	*P*
***Dams (week 9 post‐partum)***			
Body weight (g)	300.49 ± 9.45	273.95 ± 7.12	*P* < 0.05
Fat* (g)	21.7 ± 0.86	18.69 ± 0.98	NS
Fat/weight ratio (%)	7.03 ± 0.22	6.80 ± 0.26	NS
***Pups***			
***At birth***			
Body weight (g)	6.34 ± 0.08	6.06 ± 0.09	*P* < 0.05
***At weaning***			
Body weight (g)	55.99 ± 1.24	48.41 ± 1.05	*P* < 0.05
Fat (g)	4.81 ± 0.16	4.23 ± 0.3	NS
Fat/weight ratio (%)	8.63 ± 0.30	8.90 ± 0.62	NS
***At wk 18 PW***			
Body weight (g)	526.70 ± 11.81	475.89 ± 11.49	*P* < 0.05
Fat (g)	20.32 ± 0.95	18.75 ± 0.87	NS
Fat/weight ratio (%)	3.83 ± 0.13	3.94 ± 0.15	NS

Data are means ± SEM.

* Fat was calculated by sum of abdominal, epididymal and perirenal fat.

AAD, amino acid‐based diet; IPD, intact protein‐based diet; PW, post‐weaning; SEM, standard error of the mean.

**Table 4:** Effect of maternal diet fed during gestation and lactation on FBG, fasting plasma insulin, I/G ratio in dams at week 9 post‐partum and in offspring at birth, weaning and at week 18 post‐weaning (*n* = 5–6 per group)

Maternal diet	IPD	AAD	*P*
***Dams (Wk 9 postpartum)***			
Glucose, mM	5.31 ± 0.28	4.90 ± 0.28	NS
Insulin, ng/ml	2.28 ± 0.43	2.35 ± 0.65	NS
I/G Ratio	0.48 ± 0.14	0.58 ± 0.09	NS
HOMA‐IR*	0.55 ± 0.03	0.55 ± 0.00	NS
OGTT *(Wk 4 pp)*	533.31 ± 21.54	444.84 ± 17.47	*P* < 0.004
***Pups***			
***At birth***			
Glucose, mM	5.25 ± 0.78	5.36 ± 0.38	NS
Insulin, ng/ml	1.13 ± 0.30	1.18 ± 0.14	NS
I/G Ratio	0.23 ± 0.06	0.22 ± 0.01	NS
HOMA‐IR	0.24 ± 0.09	0.26 ± 0.05	NS
***At weaning***			
Glucose, mM	5.41 ± 0.50	4.88 ± 0.56	NS
Insulin, ng/ml	1.66 ± 0.06	1.91 ± 0.24	NS
I/G Ratio	0.33 ± 0.03	0.34 ± 0.04	NS
HOMA‐IR	0.38 ± 0.04	0.31 ± 0.03	NS
***At wk 18 PW***			
Glucose, mM	4.64 ± 0.15	5.52 ± 0.20	*P* < 0.05
Insulin, ng/ml	2.17 ± 0.42	3.32 ± 0.10	NS
I/G Ratio	0.45 ± 0.09	0.61 ± 0.21	NS
HOMA‐IR	0.42 ± 0.85	0.72 ± 0.24	NS

Data are means ± SEM.

* HOMA‐IR index was calculated as fasting glucose (mM) multiplied by fasting insulin (ng mL^−1^) divided by 22.5.

AAD, maternal amino acid‐based diet; G, glucose; HOMA‐IR, homeostatic model of assessment insulin resistance; I, insulin; IPD, maternal intact protein‐based diet; NS, not significant; PP, post‐partum; PW, post‐weaning; SEM, standard error of the mean.

### Pups

**Figure 2:**
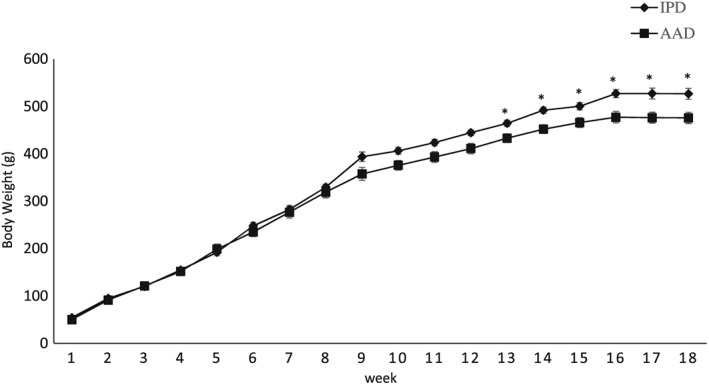
Effect of maternal diet on post‐weaning body weight of male offspring (*n* = 24 per group). Data are means ± standard error of the mean; body weight was analyzed by MIXED procedure with maternal diets and time as main factors. Time: *P* < 0.0001; maternal diet: *P* < 0.009; weaning diet: *P* = 0.06. AAD, amino acid‐based diet; IPD, intact protein‐based diet.

Birth weight was altered by maternal diet. It was higher in pups born to dams fed IPD (*P* < 0.05) (Table 3). BW was also higher at weeks 13 to 18 PW in pups born to dams fed IPD (*P* < 0.009) (Figure 2). Moreover, weaning diet had a relatively significant effect on BW (*P* = 0.06): Pups fed the IPD had relatively higher BW compared with those fed the AAD. DBP was altered by maternal diet: It was higher in pups born to dams fed the AAD compared with those born to dams fed the IPD (*P* < 0.04) (Table 5). SBP was also relatively higher in pups born to dams fed the AAD (*P* = 0.06) (Table 5). No effect of either maternal or weaning diet on pulse rate was observed. FBG was also altered by the maternal diet: It was higher in pups born to dams fed the AAD compared with those born to dams fed the IPD (*P* < 0.01) (Table 6). BG response to glucose also approached significance in pups born to dams fed the AAD (*P* = 0.06) (Table 6).

**Table 5:** Effect of maternal and weaning diets on SBP, DBP and pulse in offspring at weeks 4, 8, 12 and 16 post‐weaning

Maternal diet		IPD		AAD		*P*
Weaning diet		IPD	AAD	IPD	AAD	
	Week					
SBP	4	120.15 ± 2.94	121.03 ± 5.70	130.98 ± 5.49	120.55 ± 8.93	M: *P* = 0.06
	8	140.31 ± 3.16	137.70 ± 4.98	153.72 ± 4.24	141.95 ± 4.27	W: NS
	12	146.27 ± 3.83	139.72 ± 3.72	148.20 ± 3.46	150.46 ± 2.93	T: *P* < 0.0001
	16	144.39 ± 3.42	147.27 ± 4.49	148.29 ± 1.72	141.46 ± 5.70	
DBP	4	83.43 ± 5.44	79.15 ± 5.95	81.09 ± 4.14	73.83 ± 4.12	
	8	76.60 ± 2.94	84.16 ± 6.02	93.90 ± 5.25	94.75 ± 4.76	M: *P* < 0.04
	12	82.05 ± 4.83	85.57 ± 5.35	83.32 ± 3.95	95.98 ± 5.32	W: NS
	16	80.96 ± 5.15	83.23 ± 6.62	89.38 ± 4.72	89.45 ± 3.25	M × T: *P* < 0.04
Pulse	4	450.29 ± 7.89	458.99 ± 9.86	448.06 ± 15.12	456.93 ± 17.74	
	8	435.73 ± 6.65	440.94 ± 12.02	474.25 ± 7.92	440.89 ± 8.94	M: NS
	12	438.72 ± 11.70	427.99 ± 11.14	438.43 ± 9.06	449.39 ± 9.82	W: NS
	16	427.53 ± 9.04	427.63 ± 14.10	440.47 ± 14.50	453.83 ± 11.20	T: NS

Data are means ± SEM.

Different letters in each row are significantly different (*P* < 0.05).

AAD, amino acid‐based diet; DBP, diastolic blood pressure; IPD, intact protein‐based diet; M, maternal diet; NS, not significant; SBP, systolic blood pressure; SEM, standard error of the mean; T, time; W, weaning diet.

**Table 6:** Effect of maternal and weaning diets on FBG and BG in BG in response to glucose in offspring at weeks 4, 8, 12 and 16 post‐weaning (*n* = 6 per group)

Maternal diet		IPD		AAD		*P*
Weaning diet		IPD	AAD	IPD	AAD	
	Week					
FBG	4	5.27 ± 0.24	5.68 ± 0.33	5.97 ± 0.41	5.60 ± 0.59	M: *P* < 0.01
	8	4.95 ± 0.13	5.45 ± 0.48	5.58 ± 0.30	5.91 ± 0.26	W: NS
	12	5.70 ± 0.20	4.77 ± 0.14	5.20 ± 0.12	5.48 ± 0.56	T: *P* < 0.04
	16	5.87 ± 0.25^a^	4.73 ± 0.17^b^	5.09 ± 0.24^a^	5.95 ± 0.22^a^	M × W: NS
OGTT	4	484.86 ± 20.87	516.88 ± 19.86	453.33 ± 6.97	540.14 ± 32.13	M: NS
	8	466.18 ± 32.71	487.57 ± 20.00	445.92 ± 19.89	467.71 ± 20.24	W: *P* = 0.06
	12	439.58 ± 23.54	439.51 ± 11.26	438.58 ± 19.11	455.97 ± 26.61	T: *P* < 0.05
	16	501.74 ± 55.46^a^	469.31 ± 47.17^a^	377.08 ± 9.76^b^	516.32 ± 22.52^a^	M × W: NS

Data are means ± SEM.

Different letters in each row are significantly different (*P* < 0.05).

AAD, amino acid‐based diet; BG, blood glucose; FBG, fasting blood glucose; IPD, intact protein‐based diet; M, maternal diet; NS, not significant; OGTT, oral glucose tolerance test; SEM, standard error of the mean; T, time; W, weaning diet.

## Discussion

The results of this study support the hypothesis that characteristics of proteins fed during gestation and lactation play a dominant role in their effect on the dams and their offspring's phenotype. These results also suggest that the source‐dependent effect of proteins, as previously reported by us 18, 19, 20, can be due to individual characteristics of proteins.

The significance of the interaction of the chemical and physical structure of dietary proteins with the regulation of metabolism and food intake has received considerable attention in the past 15 years 8. The primary focus of the majority of the studies has been on understanding the role of amino acid composition and sequence, BAPs encrypted in protein's structure, digestion kinetics and also non‐protein components conjugated with proteins (e.g. calcium and phosphorous content of casein) as potential factors determining the metabolic and physiologic functions of proteins 8.

In this study, the difference between amino acid composition and content of AAD and IPDs is negligible (Table 2) with the exception of lysine that was 20% higher in AAD diet compared with IPD diet. However, to the best of our knowledge there is no evidence indicating the effect of lysine on measured parameters in this study. Therefore, our findings cannot be attributed to individual amino acids or amino acid composition of the diets. Moreover, these results must be interpreted based on the fact that both diets were standardized (AIN‐93 G‐based diets, and therefore, they are nutritionally adequate and balanced. However, the difference in digestion kinetics and also BAPs encrypted in casein (the sole source of protein in IPD) are potential factors that may play a determining role in outcomes of this study.

As previously described, the difference in amino acid kinetics may result in variations in postprandial metabolic and physiologic response to proteins 29, 30. Casein is classified as a slow protein and therefore delays amino acid delivery to the gut, while free amino acids released from AAD will be absorbed at a much faster pace compared with amino acids trapped in intact casein. This quick absorption of amino acids may have a negative impact on amino acid utilization and protein synthesis in the post‐absorptive stage 8. For example, rapid digestion and absorption of soy protein resulted in a larger portion of amino acid degradation and consequently, less protein synthesis compared with casein 31. It may explain, at least partially, lower lean mass and BW observed in dams fed the AAD in this study. However, to our knowledge, there is no study examining the role of proteins' or amino acids' digestion kinetics on fetal programming.

Casein, as a major protein in bovine milk, is a micellar phosphoprotein comprised by αs1‐casein, αs2‐casein, β‐casein and κ‐casein and known as casein micelles. Commercial preparation of κ‐casein typically contains about 5% of carbohydrates (w/w) (trisaccharides or tetrasaccharides), consisting of *N*‐acetylneuraminic acid (sialic acid), galactose and *N*‐acetylgalactosamine. κ‐casein is comprised of para‐κ‐casein (residues 1–105) and caseinomacropeptide (residues 106–169), and its glycosylated form glycomacropeptide 8. Casein also contains substantial amount of phosphorous and calcium in micellar form 8. Peptides produced during different stages of digestion of casein may express a variety of functions in the GI tract 32, 33 including the regulation of digestive enzymes, the modulation of nutrient absorption in the intestinal tract and also post‐absorptive metabolic signals. For example, glycomacropeptide from κ‐casein suppresses protein hydrolysis by inhibiting gastric secretion and motility 34. The more favourable effect of casein on blood pressure in this study can be attributed to its BAPs as it is evidenced by the fact that hydrolysates of proteins lower BP in both human and animals. Casein is rich in casomorphins, one of the major groups of BAPs in casein, capable of activating opioid receptors in the enteric nervous system and on the vagus nerve, resulting in vasodilation and lowering blood pressure 35, 36, 37. Daily consumption of casein hydrolysate (0·49 g d^−1^) containing the peptides valine–proline–proline and isoleucine–proline–proline, lowered both SBP and DBP in hypertensive subjects 38. In addition, oral administration of casein hydrolysate (32 mg kg^−1^ BW per day) decreased BP in hypertensive rats 39. Whether BAPs can cross the placenta and alter fetal development directly or indirectly by influencing maternal metabolism has not been shown.

In this study, administration of an intact protein‐based maternal diet resulted in lower FBG in offspring at week 18 PW (Table 4). The effect of proteins on glucose metabolism depends on amino acid composition, digestion kinetics of proteins and amino acid utilization in the GI tract. In general, when consumed with carbohydrates, dietary proteins reduce glycemic response 40, 41. This can be because proteins and BAPs delay gastric emptying resulting in lower glucose absorption rate and glycemic response 8. Moreover, postprandial elevation of plasma amino acids stimulates the secretion of both insulin and glucagon 42, 43 and, therefore, alters hepatic glucose metabolism by changing the portal insulin/glucagon ratio 44. However, the ultimate change in plasma glucose levels depends on the overall change in insulin/glucagon ratio.

Administration of the maternal AAD resulted in lower birth weight compared with the IPD. Although this observation is extremely important, the potential mechanisms by which maternal AAD resulted in lower birth weight is unclear at current. Low birth weight is associated with a higher risk of metabolic diseases in adulthood, including type 2 diabetes, hypertension and dyslipidemia 45, 46. It may explain the higher SBP and DBP observed in offspring born to dams fed AAD. However, lower birth weight observed in pups born to dams fed AAD was followed by a rapid catch‐up growth during lactation, which is consistent with previous studies 47, 48, 49. The negative impact on health of catch‐up growth followed by a low birth weight in later life is not clear. In some studies, rapid weight gain in the early postnatal period had a negative impact on health later in life 50, 51, 52, 53, while other studies reported that low weight at 1 year of age or low weight gain in the first year of life increased the risk of developing metabolic and cardiovascular disease later in life, independent of catch‐up growth 54, 55, 56, 57, 58, 59.

Birth weight has also been inversely associated with truncal/peripheral fat ratio but not with relative body fat at 6 months 60. This finding is consistent with our observation that in spite of lower BW in pups born to dams fed AAD at week 18 compared with those born to dams fed IPD, no difference in fat/weight % was observed.

Maternal diet had a predominant effect on various aspects of pups' health in this study, while weaning diet did not show any significant influence on measured outcomes. However, the effect of weaning diet on pups' BW approached significance (*P* = 0.06). Interestingly, AAD with relatively higher calorie density compared with IPD (3,811 and 3,597 cal kg^−1^, respectively) resulted in lower BW in both dams and offspring.

In summary, the results of this study for the first time revealed that chemical and physical structure of proteins are dominant factors influencing growth and development in both the fetal and neonatal periods. The finding that intact protein has more favourable effect on health of the offspring highlights metabolic and physiologic functions of proteins that are attributed to their physico‐chemical properties and are far beyond their traditional nutritional role as a source of indispensable amino acids and calories. Evidently, even though both AAD and IPD are nutritionally complete diets, the molecular aspects of proteins fed during pregnancy and lactation in animal models of development could be a factor influencing the phenotype of the offspring. However, this area of research is still in infancy, and much more research is needed to understand the underlying mechanisms by which proteins influence developmental programming.

## Conclusions

Our results indicate that the physico‐chemical structure of proteins fed to dams is important in altering risk factors of MS in offspring, while weaning diets do not seem to play a role. Feeding a diet composed of intact protein (casein) to pregnant and lactating dams was more beneficial than feeding a diet composed of amino acids regarding risk factors of MS. These results also support the notion that proteins exhibit many physiological functions beyond their nutritional roles. Therefore, the current dietary and clinical recommendations must be updated based on new findings in the field of proteins particularly considering their role during development.

## Conflict of Interest Statement

The authors declared no conflicts of interest.

## Funding

This study is supported by US Foundation, University of North Florida, Brooks College of Health.
